# Combining random forest with multi-block local binary pattern feature selection for multiclass head pose estimation

**DOI:** 10.1371/journal.pone.0180792

**Published:** 2017-07-17

**Authors:** Min-Joo Kang, Jung-Kyung Lee, Je-Won Kang

**Affiliations:** The Department of Electronics Engineering, Ewha W. University, Seoul, Republic of Korea; Nanjing Normal University, CHINA

## Abstract

A new head pose estimation technique based on Random Forest (RF) and texture features for facial image analysis using a monocular camera is proposed in this paper, especially about how to efficiently combine the random forest and the features. In the proposed technique a randomized tree with useful attributes is trained to improve estimation accuracy and tolerance of occlusions and illumination. Specifically, a number of features including Multi-scale Block Local Block Pattern (MB-LBP) are extracted from an image, and random features such as the MB-LBP scale parameters, a block coordinate, and a layer of an image pyramid in the feature pool are used for training the tree. The randomized tree aims to maximize the information gain at each node while random samples traverse the nodes in the tree. To this aim, a split function considering the uniform property of the LBP feature is developed to move sample blocks to the left or the right children nodes. The trees are independently trained with random inputs, yet they are grouped to form a random forest so that the results collected from the trees are used for make the final decision. Precisely, we use a Maximum-A-Posteriori criterion in the decision. It is demonstrated with experimental results that the proposed technique provides significantly enhanced classification performance in the head pose estimation in various conditions of illumination, poses, expressions, and facial occlusions.

## 1 Introduction

Head pose estimation is the front-end technique to infer the changes in view points of a human face in an image as the heading estimation is important in human navigation and locomotion [1, 2]. Many face-related computer vision systems provide the best performance to the frontal views of faces even though the human faces in an image are often non-frontal with various poses. Thus, the head pose estimation aims to facilitate the computer vision applications. In [3, 4], the faces are rotated as a result of the pose estimation to perform face recognition and face expression analysis, respectively. In [5] the frontal faces are used for retrieving key frames in video summarization. In [6] head pose information is employed for gaze estimation and human activity recognition.

The algorithms can have different granularity though they handle the same vision task. At the coarse granularity, the algorithms is applied to determine a pose among several discrete orientations, e.g. typically 5∼9 directions considering the degree of the freedom (DoF) of human heads [7–9]. At the fine granularity, the algorithms estimate the continuous angles from regression methods in the full 3D position of a head [10–12]. However, in practice, the ground truth of an accurate angle is difficult to obtain because the subject is not located at the same 3D space. For instance, Fanelli *et al*. use supplemental depth images to the estimation of a 3D position [11]. In [12, 13], Kinect sensors are used for obtaining depth information and performing the regression in 3D coordinates.

Most of the head pose estimation techniques need a series of steps to interpret a high-level understanding of orientation from the face image [14]. In other words, a statistical model is established to transform the pixel-based representation of a head to the feature subspace, followed by an optimized classifier. The algorithms needs to be robust to a variety of image-changing factors, *e*.*g*. illumination changes, facial expressions, and the occlusions with hats and glasses. In the point of the view, a number of related works have been studied in the field of the head pose estimation. In [7, 15] the high dimensional spaces of face images are mapped into the lower dimensional manifolds. In [16] the pose variation as a 3-sphere manifold is modeled in the high-dimensional feature space. Statistical distributions of face appearances, named Active Appearance Model (AAM) are developed [17, 18]. Several low-level texture descriptors are used for distinguishing the facial features in the appearance [8, 9, 19–23]. In [8] Haar-like features trained with AdaBoost are used for detecting distinctive facial features. In [13, 19, 24] a histogram of oriented gradients (HoG) descriptors are used for the face pose estimation. Local Binary Pattern (LBP)-based descriptors are widely used for the classification because they are compact and reliable to image changes. In [21, 22, 25], the LBP-based feature descriptors including Gabor feature and run-length matrix are used for representing facial features. In [25] a local directional quaternary pattern (LDQP) is proposed to represent directional changes in pixels as a variation of an LBP. In addition, deep learning based image features are used for the pose estimation, trained from a large size of face data [26, 27].

Random Forest (RF) refers to an ensemble of trained decision trees [28], shown to be effectively applied to classification problems in many computer vision applications. RF can naturally manage multiclass problems because leaf nodes in a tree correspond to classes. Each tree in the forest is independently trained with random samples, and it is combined together to construct a group of the trees, providing classification or regression. RF is also widely used for previous head pose estimation research [22, 29–33]. In the works the random forest improves the classification accuracy and the run-time efficiency as compared to the conventional approaches in the classification, *e*.*g*. PCA and SVM. The classification performance relies on how to maximize the discriminative power at each node in RF, achieved by an ability of the split function. Kim *et al*. use information gain to develop the random forest [22] with a run-length matrix of bit patterns. Huang *et al*. discriminate various head poses using Gabor features and the linear discriminant analysis (LDA) at the node test [31]. In [29, 30] the random forest regression is employed for the head pose estimation after detecting a human face. In [32] compressive features obtained from sparse responses of color and gradient components are used for random projection forest algorithm. In [33] a regression forest is trained from random face patches, which shows superior performance in the unrestricted databases. In [32, 33], the random forest is shown to be robust to in-the-wild databases by learning image samples. However, in developing the split functions, the previous works have rarely considered the characteristics of features used for the facial data abstraction. As compared to the works, the proposed technique shows how to combine the random Forest with efficient facial analysis features for the head pose estimation.

In this paper, we propose the multiclass head pose estimation algorithm at the coarse-level prediction, which uses a randomized tree incorporating an multi-block LBP (MB-LBP) to be reliable with facial occlusions. In previous works [22, 29, 31–33], the randomized trees with various image features have been introduced, yet there have been less efforts made in efficiently combining the trees and their ingredients to maximize the discriminative performance. In this work, we develop the randomized tree that includes an effective split function to learn important facial patterns represented by the LBP descriptors. Specifically, we consider the uniform property of MB-LBP in designing the split function. Furthermore several random attributes of image patches are taken into account in the construction of the random tree because the LBP-based descriptor alone may be too sensitive to local noises or occlusions. To this aim, we use Gaussian image pyramid and different sizes of block patches when encoding LPB patterns. In the classification, the trees grouped in the random forest are used for the final decision by using Maximum-A-Posteriori (MAP) criterion. It is demonstrated in the experimental results that the integration of the developed features and the random forest achieves significantly improved classification performance in various conditions of illumination, poses, expressions, and facial occlusions.

## 2 Preliminary

### 2.1 Local binary pattern applied to face analysis

**Fig 1:**
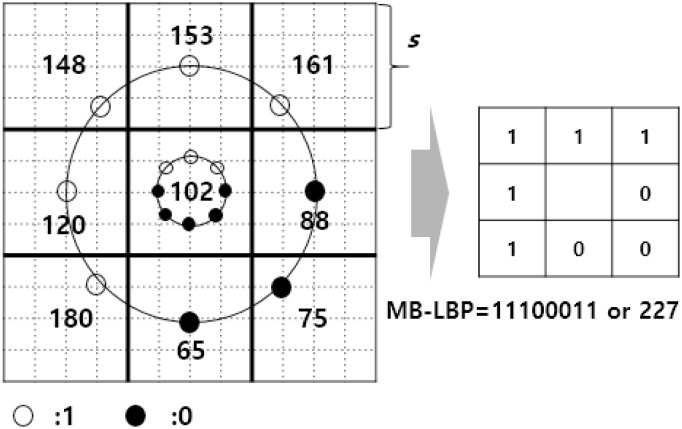
The original LBP and MB-LBP with a scale parameter *s*.

The original LBP operator assigns pixels in a 3 × 3 block into a binary string [34]. The operator compares the 8 immediately neighboring pixels to the center pixel and encodes the result as an eight-bin sequence. The LBP is robust to illumination changes because it computes the signs of pixel differences. However, the patterns may be readily distorted from the noises and small pixel variations. Therefore, the LBP is extended later in different applications [35, 36]. In one extension, the LBP operator is applied to the surrounding blocks at different scales, named multi-scale block LBP (MB-LBP) [36]. The multi-resolution analysis of a block uses the average values of surrounding sub-blocks when comparing those to the center block. Fig 1 shows the original LBP and the MB-LBP when the size of the sub-block is 4. In Fig 1 the pixel values are the averages of the sub-blocks, and “1” is assigned if the corresponding neighborhood is greater than or equal to the center value. Otherwise, “0” is assigned. The binary sequence created by MB-LBP is “11100011” (or 227 as a decimal number) in the example.

**Fig 2:**
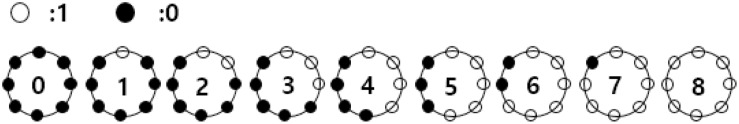
Nine uniform LBP patterns.

The number of the possible LBP patterns can be too many as shown in the example, and the high dimensional feature space may incur an over-fitting problem in learning. Thus, in another extension, a sub-group of the LBP patterns, named a uniform LBP, is considered to resolve the problem. The uniform LBP is defined as a binary string that includes at most two bitwise transitions from 0 to 1 or vice versa in the circular presentation as shown in Fig 2. The uniform LBP shows several useful properties. First the nine spatial micro-structures are used for representative patterns, including a bright spot (0), edges and corners (1∼7), and a homogeneous region (8) because they are frequently appeared in the textures. In [35, 36], it is observed that the uniform patterns account for around 90% of all LBP patterns in facial data while only the 58 patterns are uniform among 256 8-bit patterns. Second the uniform LBP is invariant in rotation, so the similar patterns can be compactly represented. Thus, considering the properties, the uniform LBP patterns can be used for a feature reduction of LBP.

The LBP operator has been widely used in facial data analysis [35, 37–40] because important facial features (e.g. a nose and eyes) incorporating distinctive micro texture patterns are well described by such operators. They consider the local descriptions of faces based on LBP features and combine them into global descriptions to be robust against pose and illumination variations. The local descriptor and the global descriptor intend to capture the micro-patterns of textures and some invariant properties, respectively. In [35] the facial image is divided into several sub-blocks where the LBP descriptors are extracted independently, and then they are linked together to the global descriptor of the face. The different sizes of the sub-blocks are used for the multi-resolution analysis of a facial image.

### 2.2 Review of random forest

**Fig 3:**
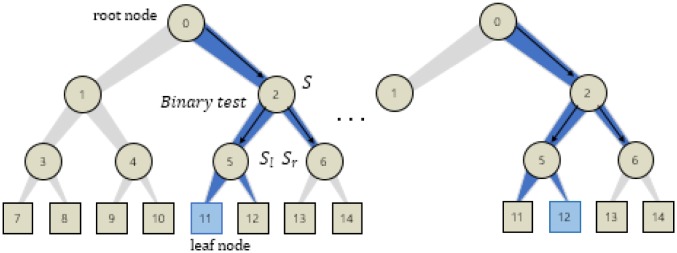
Randomized trees including a root node, internal nodes, and leaf nodes and edges. The random forest consists of the trees.

In this section we review the training and testing of a random forest. Random forest (RF) turns out to be an efficient machine learning technique in many computer vision applications. It is shown in [28] that a group of randomized trees provides high generalization power while the decision tree alone may suffer from an overfitting problem. Thus, the random forest is formed with an ensemble of the trees as shown in Fig 3. Furthermore, to achieve the generalization, the trees are built with considering randomness in training. The training samples are randomly chosen either for growing the tree, for optimizing the node decision, or for the both.

A tree *T* in the forest $T=\{T_{i}\}$ consists of several nodes including a root node, internal nodes, and leaf node, and edges connecting with the nodes, shown in Fig 3. Learning a randomized tree is supervised, *i*.*e*., the training samples are annotated with label information. In the training, the goal is to maximize the classification performance when input samples traverse from a root node to a leaf node corresponding to each label. To this aim, each internal node needs to make its own optimal binary decision using a split function, formulated *e*.*g*. in the input data $v\in R^{d}$ arriving at the *i*-th node,

$$\begin{array}{c} h_{\phi }\left(v,\phi_{i}\right):R^{d}\times P\to \left\{0,1\right\}, \end{array}$$ (1)

where *ϕ*_*i*_ denotes the split parameters associated with the *i*-th node in the set of all split parameters P, and 0 and 1 are understood as the left and the right children nodes to be placed.

There are several research works developing the binary tests in the pose estimation. Li *et al*. use the the pixel intensities at two different pixel positions [29]. Huang *et al*. apply linear discriminative analysis to the test [31]. However, the information gain (IG) is useful in general cases [28]. In information theory, IG is defined as the reduction of uncertainty when the training data arriving at the current node is divided into the children nodes. IG is mathematically defined as:

$$\begin{array}{c} IG=H\left(S_{i}\right)-\sum_{j\in L,R}\frac{|S_{i}^{j}|}{|S_{i}|}H\left(S_{i}^{j}\right), \end{array}$$ (2)

where *S*_*i*_ refers to the data set at the *i*-th node being split into the two subsets *S*^*L*^ and *S*^*R*^ in the left children and in the right children, respectively. *H*(*S*) is the entropy.

In testing, an unseen sample traverses the tree down to a leaf node by using the trained split functions with the associated parameters. The input sample is accordingly moved either to the left child node or to the right child node. The estimation is done when the sample is stopped to a leaf node. Note we construct a group of trees in the random forest. Therefore the final decision is made by considering the results from all the trees.

## 3 Proposed technique

**Fig 4:**
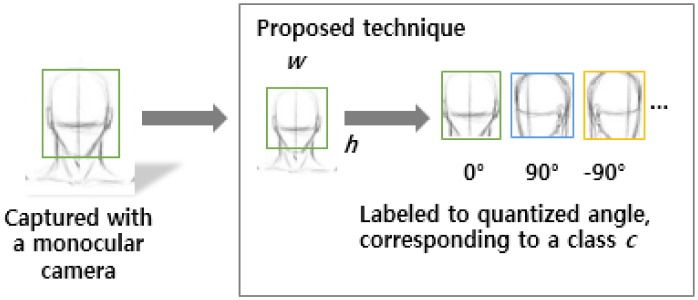
The processing pipeline of the head pose estimation in the proposed technique.

There are two subsequent tasks in the head pose estimation, *i*.*e*., the face detection in an image and the following pose estimation. In this paper, we assume facial data would be already localized for the pose estimation, and focus on the latter problem, as shown in Fig 4. Facial images obtained from monocular cameras are detected and cropped with face detection algorithm such as Viola-Jones method. For this, we use a standard facial image set named CMU Multi-PIE [41], including various face orientations, illumination conditions, and facial expressions, to resolve the problem. The image sets are annotated with pre-defined rotation angles that are quantized (e.g. 5∼9) based on the degree-of-freedom of human faces [14].

### 3.1 Proposed feature space

In the proposed technique, a facial image is normalized to *W* × *H* size. Specifically *W* and *H* are equal to 108. Then, a gaussian image pyramid that is a sequence of low-pass filtered images of an original image is applied as a pre-processing step to an image patch. Because the LBP features can be too sensitive to local noises or occlusions, a gaussian image pyramid is applied to the input images before the feature extraction. The original image denoted by *G*_0_ is sequentially filtered with a Gaussian kernel *w* whose filter tab is 11 × 11 and the standard variation is set to 1. Then, the images are sub-sampled by a factor of two to generate the sequence of reduced resolution images *G*_*l*_. The levels of the pyramid are obtained iteratively. Mathematically, they are given as,

$$\begin{array}{c} G_{l}\left(i,j\right)=\sum_{m}\sum_{n}w\left(m,n\right)G_{l-1}\left(2i+m,2j+n\right). \end{array}$$ (3)

In the proposed technique *l* is set to 0, 1, and 2. As the size of a facial image is normalized to 108, corresponding to *G*_0_, the next layered images corresponding to *G*_1_ and *G*_2_ are equal to 54 and 27, respectively.

**Fig 5:**
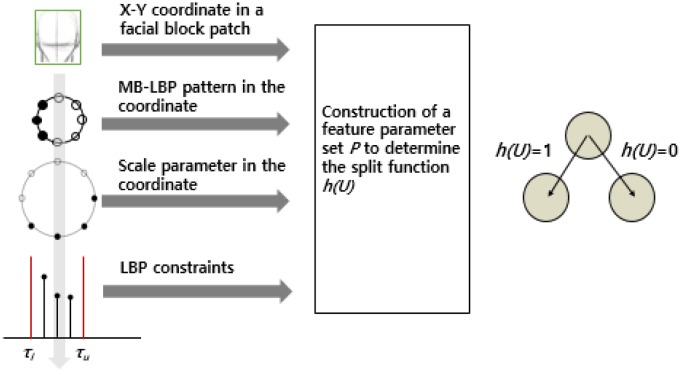
MB-LBP based feature set including the size of the block *s*, the center position *k*, and the scale of the Gaussian image pyramid *g*, and the upper and the lower thresholds, which are used for establishing a split function in each node of a randomized tree.

*M*_*g*,*s*,*k*_ denotes an MB-LBP feature obtained from a randomly chosen block in an image. In *M*_*g*,*s*,*k*_,*s* refers to a block size, which can be either 1, 4, 12, or 36. Thus, there are four MB-LBP feature spaces. *g* refers to a level of an image pyramid, which can be either 0, 1, and 2. *k* is the center pixel position of an MB-LBP block to retrieve the bit-pattern. The blocks can overlap one another during the feature extraction, so *k* can be any pixel position in a block if the block is fully contained in the image. Fig 5 shows the proposed feature set. The features are used for constructing a feature set *p* in all possible parameter space *P*.

The number of all the possible MB-LBP patterns is too large, which may cause an overfitting problem by the high dimensional feature spaces. Therefore we quantize the MB-LBP to a uniform MB-LBP denoted by *U*_*g*,*s*,*k*_ for a feature reduction. Among all the possible uniform MB-LBP patterns, *U*_*g*,*s*,*k*_ is formed as the closest bit-pattern from *M*_*g*,*s*,*k*_ with respect to the Hamming distance. For example, “11100101” is converted to “11100111.” If there are multiple candidates, the less significant bits are changed. It is observed in the facial image data that the uniform patterns are more than 90% of the LBP patterns while only the 58 patterns are uniform. Thus, we employ the properties for the feature reduction in the training.

### 3.2 Proposed random forest

Optimizing the split function is important in the developments of the random forest. The function needs to be tailored to the MB-LBP based features. For this, we propose a split function *h*(.) for *U*_*g*,*s*,*k*_ to be trained in a randomized tree *T*, defined as,

$$\begin{array}{c} h_{g,s,k,\tau_{l},\tau_{u}}\left(U_{g,s,k}\right)≔\{\begin{array}{cc} 1, & \;2^{\tau_{l}}<U_{g,s,k}<2^{\tau_{u}}\\ 0, & \;oherwise \end{array} \end{array}$$ (4)

where *U*_*g*,*s*,*k*_ is the uniform MB-LBP with a level of an image pyramid *g*, a scale parameter *s*, and a position *k*. *τ*_*u*_ and *τ*_*l*_ are two constraint thresholds that are, respectively, used for the upper bound and the lower bound of decimal representation of the uniform MB-LBP. All the parameters are exemplified in Fig 5. It is highlighted that the two constraints regarding the upper bound and the lower bound are used for compactly clustering the similar textures because there are at most two bit transitions in a uniform LBP. The selected parameter set is trained to determine the split function *h*, as shown in Fig 5.

The function is to map an input *U*_*g*,*s*,*k*_ to the binary outputs 0 and 1. Based on the binary test, the training samples at a node in a randomized tree are split into two children nodes. If the output of the function is true, the samples are sent to the left child node. Otherwise, they are sent to the right child node. The parameters at the nodes are learnt during the training to maximize an objective function. We employ the information gain function [28] that is defined as the difference between the differential entropy of the parent node and the sum of the differential entropies of the children nodes. The idea behind is that the information gain increases more when a child node contains less diversified classes, thus more discriminative capability of the tree. information gain function *I* is given as,

$$\begin{array}{c} I\left(g,s,k,\tau_{l},\tau_{u}\right)=H\left(U_{g,s,k,\tau_{l},\tau_{u}}\right)-\sum_{i\in L,R}\frac{|U_{g,s,k,\tau_{l},\tau_{u}}^{i}|}{|U_{g,s,k,\tau_{l},\tau_{u}}|}H\left(U_{g,s,k,\tau_{l},\tau_{u}}^{i}\right), \end{array}$$ (5)

where *H*(*U*) is an entropy to measure uncertainty. The entropy in the proposed technique is defined as *H*(*U*) = −∑_*c*∈*C*_ *p*(*c*|*U*) log *p*(*c*|*U*) where *C* is the set of classes and *p* is a probability of samples with a label *c* at a node specified by *U*. The distribution of the classes in the left and the right children is changed by *U* at a node, and the number of the classes is counted to compute the distribution. The information gain increases more if a child node has less diversified entries. Thus, the optimal parameter $\left(g^{*},s^{*},k^{*},\tau_{l}^{*},\tau_{u}^{*}\right)$ in the split is given as,

$$\begin{array}{c} \left(g^{*},s^{*},k^{*},\tau_{l}^{*},\tau_{u}^{*}\right)={\arg\max}_{g,s,k,\tau_{l},\tau_{u}\in P_{j}}I\left(g,s,k,\tau_{l},\tau_{u}\right), \end{array}$$ (6)

where *P*_*j*_ is the randomly chosen parameter space in all possible set *P* at the *j*-th node.

**Fig 6:**
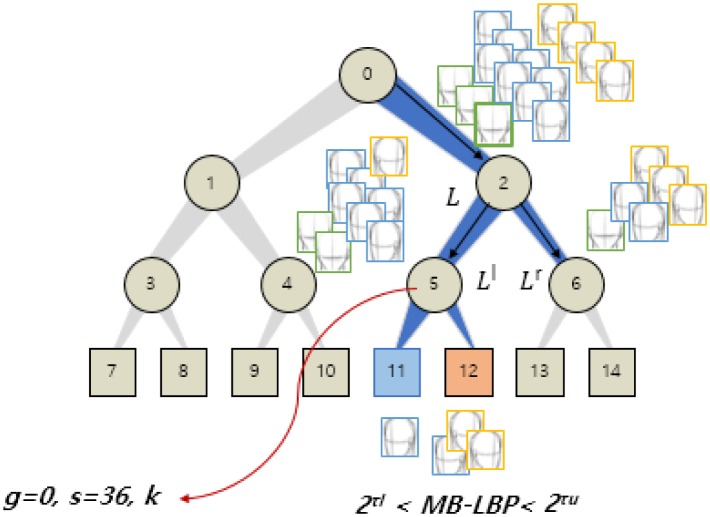
Construction of a randomized tree.

The optimized parameters are stored at internal nodes while constructing a randomized tree in the training. For example, in Fig 6, the optimal parameters maximizing the information gain are used in the node 5. The same optimization is repeatedly performed at each subsequent node. We also use a bagging that extracts random training samples from the image set for each tree. The bagging allows reliable performance results against large variants of input data while using less memory sizes in training. The training stops when the termination condition satisfies. In the standard RF training [28], the tree stops growing if it reaches to the pre-defined maximum depth, or if there are too few samples remaining in the current node. Specifically we set the maximum depth of a tree and the minimum samples in a node, respectively, to 9 and 5. We will show experimental results with respect to various termination conditions in the experimental results.

Training a randomized tree is to build each optimal weak classifier corresponding to a node in the tree structure. On top of that, the tree also needs to provide an accurate prediction model at the leaf nodes. In the supervised learning, a subset of labeled training samples is associated with leaf nodes, and therefore the distributions of the labels can be used for the prediction. Precisely, we employ the conditional distributions after observing the associated samples, *i*.*e*., *p*(*c*|*u*) where *c* is the label of the head pose class in all possible set *C*, *u* is the uniform MB-LBP sample. Subsequently, we use a Maximum-A-Posteriori (MAP) for the predictor, defined as

$$\begin{array}{c} c^{*}=\arg\max_{c\in C}p\left(c|u\right). \end{array}$$ (7)

For instance, in Fig 6, the node 11 and the node 12 are chosen, respectively, for the left and the frontal faces as they are major in the leaf nodes.

In a testing, a previously unseen sample traverses the tree down to a leaf node by going through the trained nodes. The split function at a node directs the samples either to the left child node or to the right child node, and, accordingly, the sample reaches to a leaf node. The estimation is done in the leaf node. Note that each randomized tree is grouped into a random forest. Therefore, in testing, all the prediction results need to be combined into a single forest prediction to make the final decision. The decision could be made with maintaining the whole conditional probability distributions. However, we use the major voting of the prediction results in the final decision as we compute the MAP prediction in a tree.

## 4 Experimental results

### 4.1 Test condition

In this section, the performance of the proposed technique is quantitatively evaluated. The experiments are performed using CMU Multi-PIE head pose image database [41], including 3,600 face images with 20 subjects with various face poses, lightening conditions, and facial expressions, controlled in a laboratory. We also use the AFLW [42], AFW [43], 300W [44], and Pointing04 [45]. AFLW, AFW, and 300W data bases are “in-the wild” data bases, and Pointing04 is another data base acquired from a laboratory condition. It is noted that any pre-processing technique to resolve the lightening variation is not applied to clearly show the performance of the proposed technique. Readers who are interested in the effects of the pre-processing such as histogram equalization may refer Tan’s work [46]. We use the Viola-Jones method to detect the faces, and the image samples are resized to 108 × 108. In prior arts [47, 48] an alignment process of a face sample has played important roles in pose estimation. In [47], a partial least squares regression-based method is used for reducing sensitivity to misalignment, thus providing better classification results. In our experiments, we use an alignment algorithm for the LBP-based descriptors to cope with geometric invariance. The facial feature points such as corners of the eyes and the tip of the nose are aligned in the samples by using trained feature sets, as in [49]. The process is automatically applied to all the facial samples that are used in the experiments.

The experiments are configured to predict the head rotation angles quantized into 3∼9 classes, equally-spaced from −90 to 90 degrees. Some of the subjects are occluded with glasses or hairs, which are used for demonstrating the reliable performance of the proposed technique against an occlusion. In training, we use 5-fold cross validation to avoid any over-fitting. For training the randomized tree, the maximum depth of tree is set to up to 9, and the minimal number of samples processed in a node is 5 to stop the tree growing. We train maximum 15 trees to create a forest. The parameters are empirically set to maximize the performance. In testing, the performance is evaluated by averaging the results in five times.

We perform the intra-data base experiments and inter-data base experiments. In the intra-data base experiments, two disjoint sets of facial samples from the same data base are separately used for training and testing. Specifically, the CMU MultiPIE data base is used for the intra-data base experiments. In the inter-data base experiments, the facial samples from the different data bases are separately used for training and testing. Specifically, the random forest is trained with the CMU MultiPIE data base, and then the trained model is tested using in-the-wild data bases [42–44] and Pointing04 acquired in laboratory conditions [45]. The results of the inter-data base experiments are shown in Sec. 4.2.4. All experiments are performed with an Intel i7 @ 3.60GHz CPU and 8GB memory.

### 4.2 Performance evaluation

#### 4.2.1 Performance comparisons to conventional techniques

**Fig 7:**
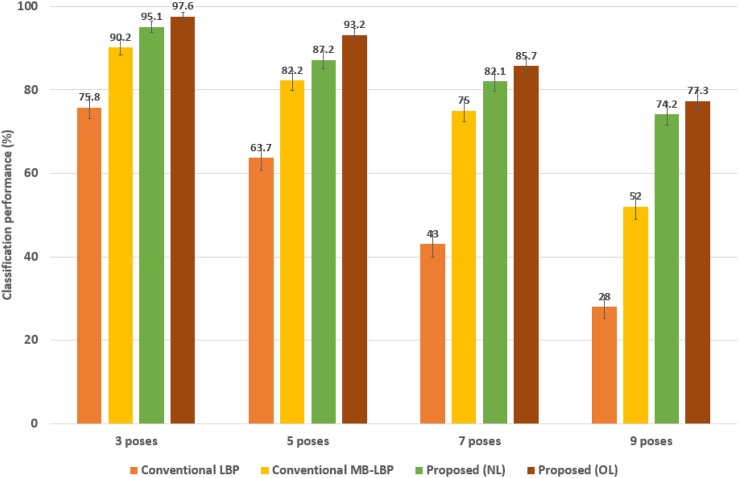
Estimation accuracies of the proposed technique with respect to the number of the classes, as compared to the conventional algorithms. Proposed (NL) refers to the technique where the uniform MB-LBP is extracted from non-overlapped block patches in the proposed technique while Proposed (OL) uses overlapped block patches in the generation. The error bars represent 95% binomial confidence intervals.

In this subsection, we present the results of the intra-data base experiments using the MultiPIE data base. We show the estimation accuracies of the proposed technique and the conventional techniques named “Conventional LBP” and “Conventional MB-LBP” with respect to the classes of the different head poses in Fig 7. “Conventional LBP” and “Conventional MB-LBP” refer to the algorithms using only the original LBP and the MB-LBP, respectively, combined with the same random forest classifiers. However, in the conventional algorithms, only one constraint parameter, *i*.*e*., *τ* of the split function is used [21]. In other words, in Eq (1), *h*_*ϕ*_(*U*_*ϕ*_) is true if a uniform MB-LBP *U*_*ϕ*_ is less than a single threshold *τ*, otherwise, it is false. Thus the performance difference shows mostly the impact of the proposed split function design on the estimation accuracy. As shown in Fig 7 the proposed technique provides significantly improved estimation accuracies over the conventional algorithms in all the numbers of the head poses. The proposed technique provides the performance about 95%, 87.2%, 82%, 74%, respectively, in 3, 5, 7, and 9 pose cases. The average performance is 85%. As compared to the average, “Conventional LBP” and “Conventional MB-LBP” provide the average performance of 53% and 75%, respectively. Even though the classification performance is monotonically degraded with the number of the classes, the performance of the proposed technique is more gentle in the degradation than the conventional techniques because of the extended block sizes in the feature extraction. For instance, Fig 7 shows 95 ∼ 74% in “Proposed (NL)” while showing 90.2 ∼ 52% in “Conventional MB-LBP”, and 75.8 ∼ 28% in “Conventional LBP”, which is much unreliable. “Conventional MB-LBP” is comparable with “Proposed (NL)” in 3 and 5 poses. However, the differences in the performance become large in 7 and 9 poses about 7∼ 22%. We show the binomial confidence interval for 95% confidence in Fig 7. The error bar represents how much uncertainty the proposed technique has in the estimation. The ranges of the errors in the proposed techniques are around ±0.9%∼±1.6%, while those in the conventional LBP-based techniques are around ±1.7%∼±3.1%.

Furthermore we provide two variations of the proposed techniques, depicted as “Proposed (NL)” and “Proposed (OL).” The candidates of the block positions to extract the uniform MBLBP features are only the differences between the algorithms. “Proposed (NL)” extracts the uniform MB-LBP features from non-overlapped *s* × *s* blocks in the image sample. In other words, the pixel position *k* in Eq (4) can be placed only on the grid of the image sample. As compared, in “Proposed (OL)”, the pixel position *k* can be any position in a block if the uniform MB-LBP feature is available. In implementation, we choose a subset of the overlapping *s* × *s* blocks during the training rather than to use all the possible pixel positions. As shown in Fig 7 the average classification performance of “Proposed (OL)” is better than that of “Proposed (NL)” about 2.5 ∼ 5.0%. Meanwhile the training time increases about 180% in “Proposed (OL)” because there can be more pixel positions, randomly selected in training the randomized tree. However, the test time is only slightly changed. Once the node parameters are determined, the classification is very quick, which is an important advantage of the random forest.

**Fig 8:**
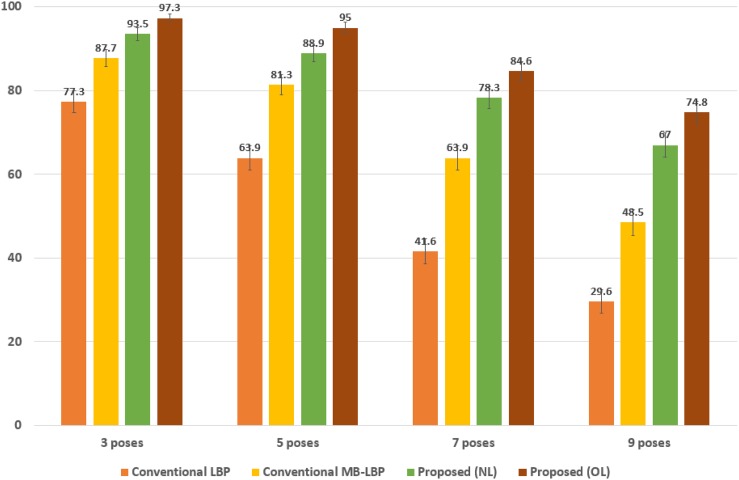
Estimation accuracies of the proposed technique with respect to the number of the classes, as compared to the conventional algorithms when the facial images have occlusions. The error bars represent 95% binomial confidence intervals.

To show the reliable discriminative power to the occlusions, we reorganize the CMU MultiPIE database to include only the faces having occlusions such as hairs and glasses, and show the results in Fig 8. The performance of the proposed technique significantly outperforms those of the two other conventional algorithms in all the number of the poses as well. The average performance of the proposed technique (*i*.*e*., “Proposed (NL)”) is 82%, which is better than those of the other two conventional techniques, *i*.*e*. 53% and 70%, respectively in “Conventional LBP” and “Conventional MB-LBP”. As shown, the performance of the proposed technique is more reliable to the occlusions than those of the conventional techniques. It varies from 93.5% in 3 pose to 67% in 9 pose, *i*.*e*., the difference among the poses is about 26.5%. However, the differences in “Conventional LBP” and “Conventional MB-LBP” are about 47.7% and 39.2%, respectively. “Proposed (OL)” yields the highest classification performance about 88% on average. We also show the binomial confidence interval for 95% confidence in Fig 8. The ranges of the errors in the proposed techniques are around ±1.1%∼±2.7%. The ranges are slightly larger than in Fig 7 as the occlusion gives higher variability in the inputs.

**Fig 9:**
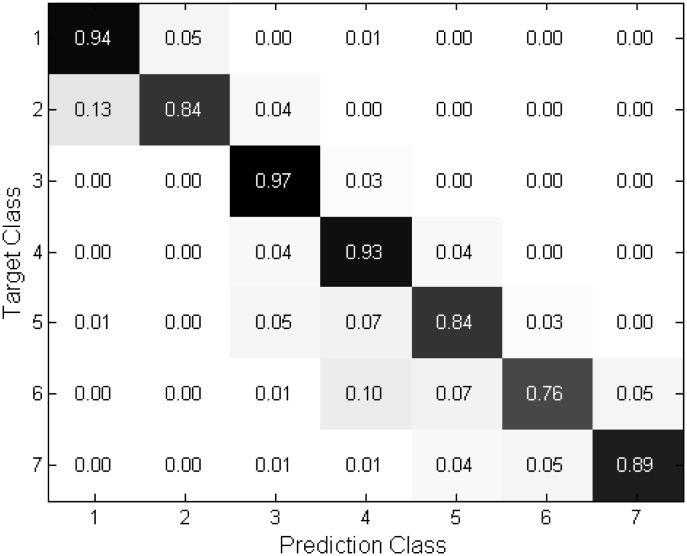
Confusion matrices of “Proposed (NL)” in 7 class case.

**Fig 10:**
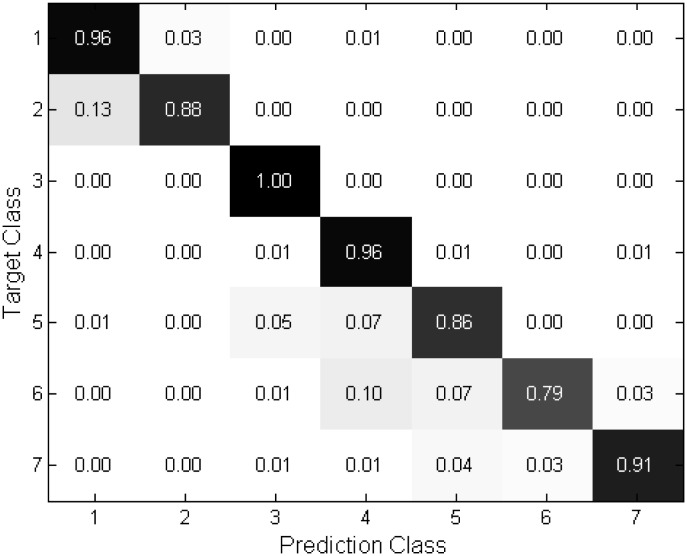
Confusion matrices of “Proposed (OL)” in 7 class case.

**Fig 11:**
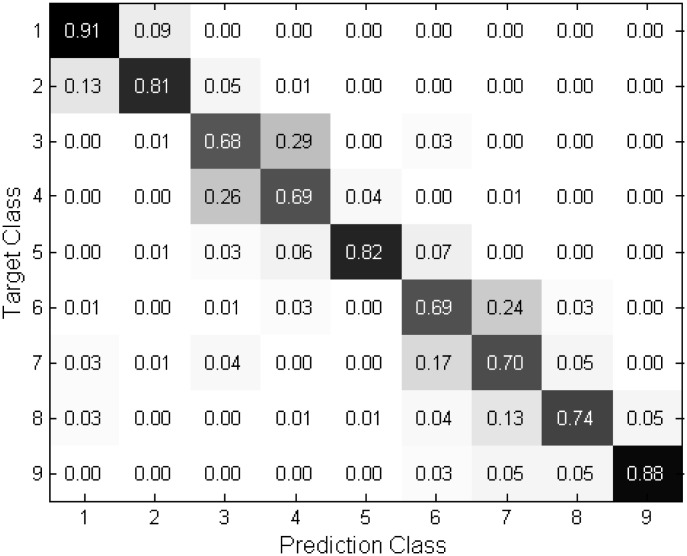
Confusion matrices of “Proposed (NL)” in 9 class case.

**Fig 12:**
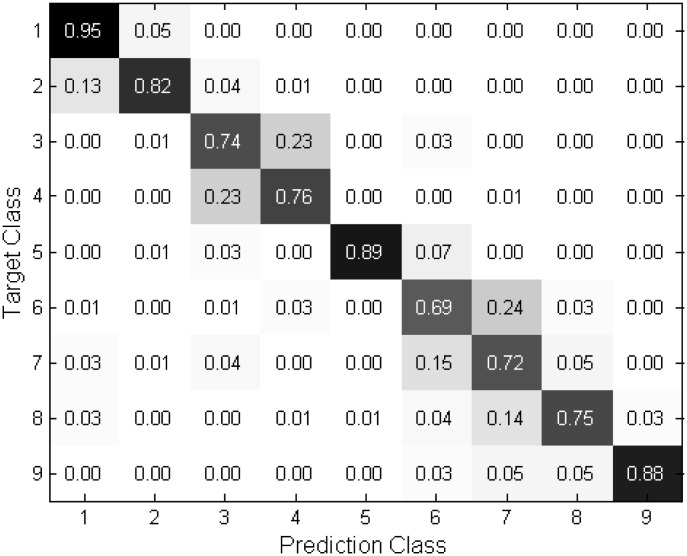
Confusion matrices of “Proposed (OL)” in 9 class case.

Several confusion matrices obtained from in 7 and 9 poses are shown in Figs 9∼12 to provide a more comprehensive analysis of the proposed technique. The matrices show that the proposed technique yields reliable performance to the estimation because most of the errors occur in neighboring angles. It is observed from Figs 11 and 12 that the performance is quite robust in estimating the frontal face and −90 and 90 degrees, corresponding the class 5, 1, and 9, respectively. However the misclassification is relatively frequent in the intermediate angles. As compared to the 9 poses, Figs 9 and 10 depict in the 7 poses that the errors are evenly distributed at the most of the classes.

#### 4.2.2 Performance analysis in various conditions on parameters

**Fig 13:**
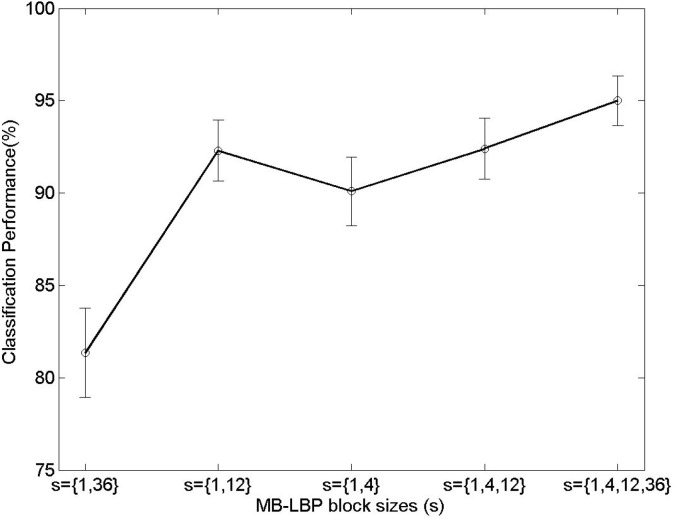
Performance changes with respect to the number of the MB-LBP feature planes. *s* refers to the size of the MB-LBP block. The error bars represent 95% binomial confidence intervals.

The proposed technique incorporates various factors that can affect the overall performance. For the purpose of experimental analysis on the factors we first change the MB-LBP parameters. The proposed technique extracts four MB-LBP feature planes (*i*.*e*. the block sizes are either 1, 4, 12, or 36) for possible candidates in training while the compared algorithms do only few number of the features. We examine the proportions of the MB-LBP sizes, selected as the best feature at each node in a random forest to figure out which sizes affect the performance. We observe from the empirical results that the proportions of the blocks are 65.5%, 11.1%, 14.8%, 8.6%, respectively for the size 1, 4, 12, and 36 in training, as shown in Table 1. In other words, the block size equal to 1 is largely selected among the candidates to maximize the information gain in the tree, and, thus we include the block size equal to 1 in all the comparisons. Fig 13 shows the classification performance with respect to the MB-LBP sizes *s* in the 5 pose case. The performance shows 81.3% when 1 × 1 and 36 × 36 block-sized MB-LBP are used. As compared, the performance is close to the best when 1 × 1 and 12 × 12 block-sized MB-LBP are used. It is noted that the 4 × 4 block size provides slight changes to the performance. The phenomenon is because the features from 4 × 4 block size may be similar to 1 × 1 block size in the second level of the Gaussian pyramid. However all the block sizes somehow contributes to improving the overall performance as revealed in Table 1. The proposed technique achieves the best performance when all the block sizes are used in the random forest.

**Table 1:** Proportions of MB-LBP block sizes, selected as the best feature at each node in a random forest.

MBLBP size(*s*)	*s* = 1	*s* = 4	*s* = 12	*s* = 36
Proportion (%)	65.5%	11.1%	14.8%	8.6%

**Fig 14:**
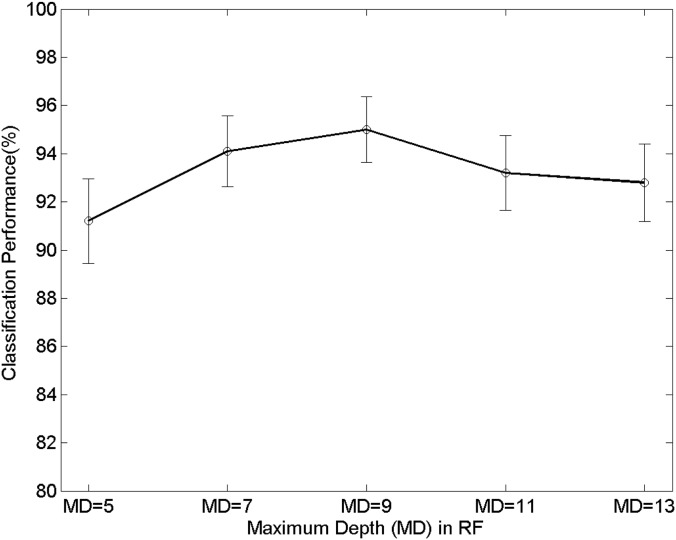
Performance changes with respect to the number of the maximum depth (MD) of the random tree. The error bars represent 95% binomial confidence intervals.

**Fig 15:**
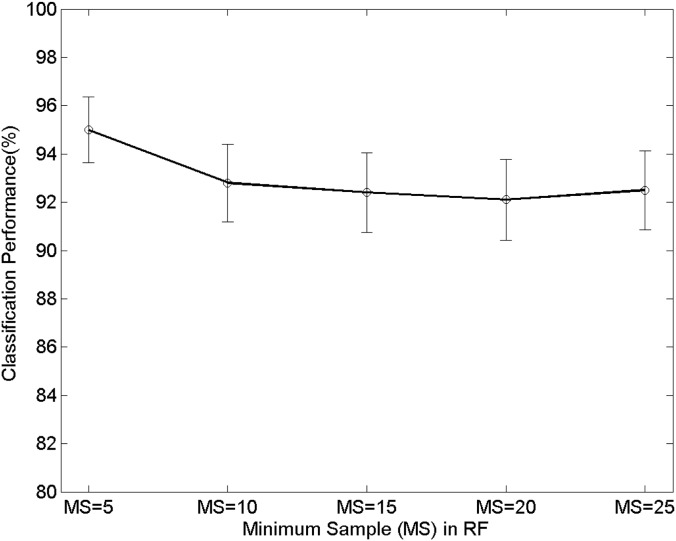
Performance changes with respect to the number of the minimum samples (MS) in the tree. The error bars represent 95% binomial confidence intervals.

**Fig 16:**
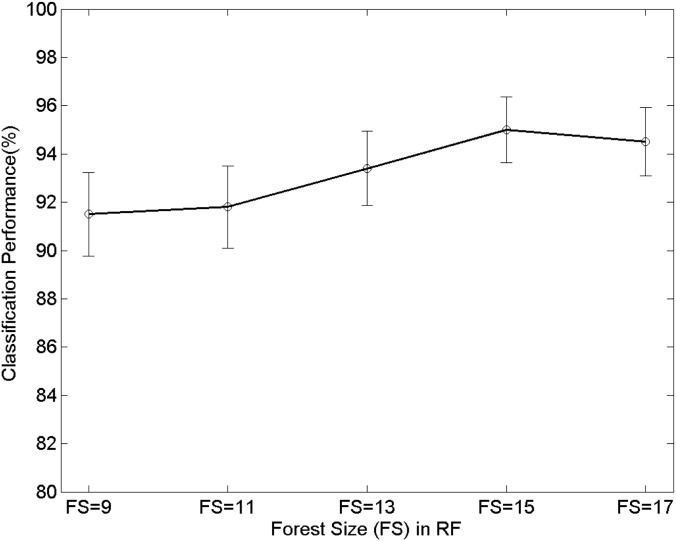
Performance changes with respect to the number of the forest size (FS). The error bars represent 95% binomial confidence intervals.

Second, the performance of the proposed technique can rely on the different parameters of a random forest, and therefore we present the effects of the changes of the parameters. Precisely, the parameters that are the maximum depth (MD) of a tree, the minimum samples (MS) of a node, and the forest size (FS) are changed. The MD and MS are used for the early-termination condition in training as a random tree finishes its growth when the maximum depth or the minimum samples reaches to the pre-defined values. Figs 14∼16 show the variations of the performance with the RF parameters MD, MS, and FS. In Fig 14, the proposed technique shows 91.2%, 94.1%, 95%, 93.2%, and 92.8% when the maximum depths (MD) are 5, 7, 9, 11, and 13, respectively. In Fig 15 the proposed technique shows 95%, 92.8%, 92.4%, 92.1%, and 92.5% when the minimum samples (MS) are 5, 10, 15, 20, and 25, respectively. The forest size (FS) determines the number of trees comprising a forest. Each tree performs the classification in training/testing independently, and each of the result is combined to make the final decision. Fig 16 shows the variations of the performance with respect to the forest size. The performance is 91.5%, 91.8%, 93.4%, 95%, and 94.5%, respectively when the sizes are 9, 11, 13, 15, and 17. We emphasize from the results that the variations of the classification performance are relatively small even though the RF parameters are different. Furthermore, the confidence intervals with respect to the different parameters are similar one another. This phenomenon highlights the robustness of the performance of the proposed technique over various conditions and practical advantages because subtle changes in the implementation do not affect significant changes in the performance.

#### 4.2.3 Performance comparison with various feature descriptors

In this subsection we show the performance of the proposed technique as compared to previous research works using various feature descriptors. For this we choose the state-of-the-art methods using different image descriptors such as histogram of gradient (HoG) feature [13, 19, 24], Gabor feature [21], and bit-pattern run length (BPRL) feature [22]. Support vector machine (SVM) is used for a classifier in [13, 19, 21] while the random forest (RF) is used for [22, 24] as in the proposed technique. We select the compared algorithms using monocular cameras processing RGB color images but also some of the algorithms use supplemental depth images, obtained from Kinect sensor [13, 19]. Some of the algorithms perform the regression of the head poses [13, 24]. In the comparison, we choose a specific angle in the regression to evaluate the performance.

**Fig 17:**
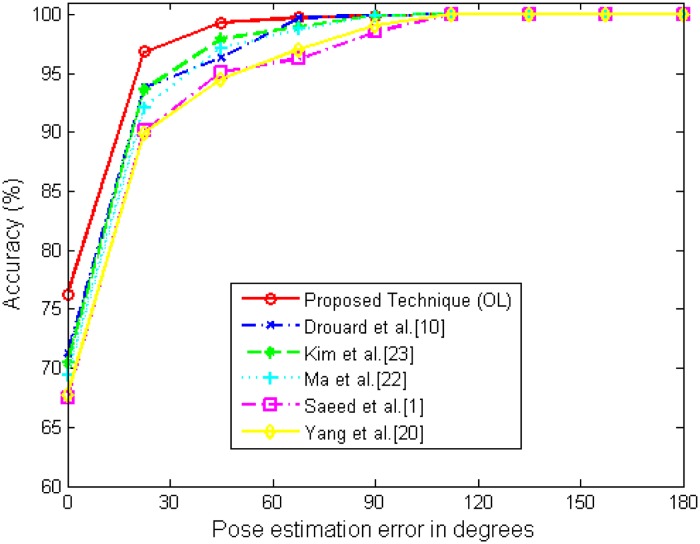
Cumulative head pose estimation error (%) of test images with respect to a degree.

Table 2 shows the results of using various image descriptors and classifiers for the head pose estimation. We observe from the results that the LBP-based descriptors provides superior performance as compared to the HoG-based descriptors. For instance, Ma *et al*. [21] use a Gabor-filtered LBP followed by SVM, providing better classification performance than the HoG-based descriptors with SVM [13, 19]. The MB-LBP based descriptors yield more robust descriptors against occlusions and illumination variants in face analysis. However, the performance relies on the classifier as well. Drouard *et al*. [24] show fairly good performance with HoG-based descriptors with the random forest. Furthermore the random forest achieves better performance with MB-LBP than with HoG, when seeing the performance of the proposed technique and the compared algorithms. The MB-LBP can provide higher generalization capability in the parameter selections. Accordingly, the proposed technique shows the best classification performance, *i*.*e*, classification error around 5.0% with 0.8% of 95% confidence interval and the mean absolute error around 4.17. The depth information enhances fair performance, observed from [13] and [19]. However, they need RGB+D camera sensors. We also show the cumulative head pose estimation error distributions (%) of test images with respect to a degree in Fig 17. As shown in Fig 17 the proposed technique provides robust classification performance in errors.

**Table 2:** The classification errors (CE), the mean absolute errors (MAE) in degree of the head pose estimation algorithms using different features and classifiers in intra-bases experiments, and the standard deviation (STD) of the degrees.

Method	Feature/Classifier	CE	MAE(degree)	STD
Ma *et al*. [21]	LGBP/SVM	10.8	5.22	7.61
Kim *et al*. [22]	BPRL/RF	9.5	5.17	6.82
Drouard *et al*. [24]	GLLiM+HoG/RF	8.2	4.83	6.75
Yang *et al*. [19]	HoG/SVM	12.1	5.35	7.50
	HoG+Depth/SVM	8.3	4.81	6.49
Saeed *et al*. [13]	HoG/SVM	12.4	5.37	7.31
	HoG+Depth/SVM	8.9	4.96	7.05
MB-LBP+SVM	MB-LBP/SVM	15.8	5.84	9.07
Proposed (NL)	MB-LBP/RF	10.6	5.20	7.13
Proposed (OL)	MB-LBP/RF	5.0	4.17	6.59

**Fig 18:**
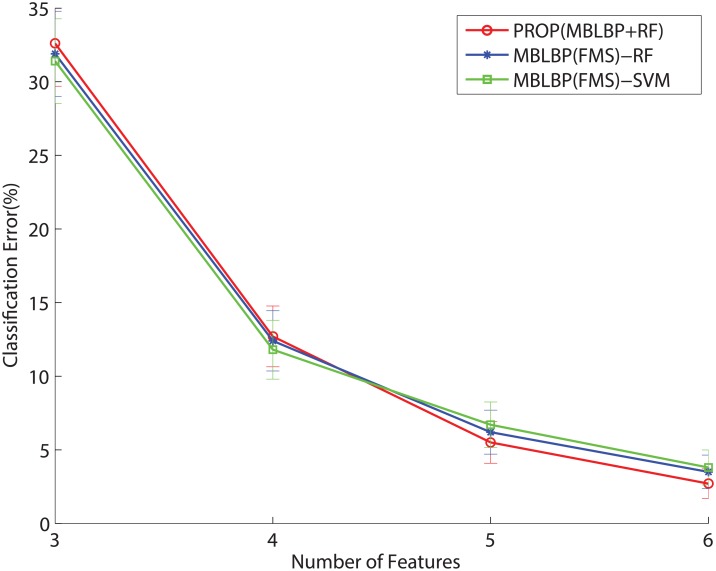
Performance changes with respect to the number of the MB-LBP feature in 3-pose case, using different features selectors and classifiers.

**Fig 19:**
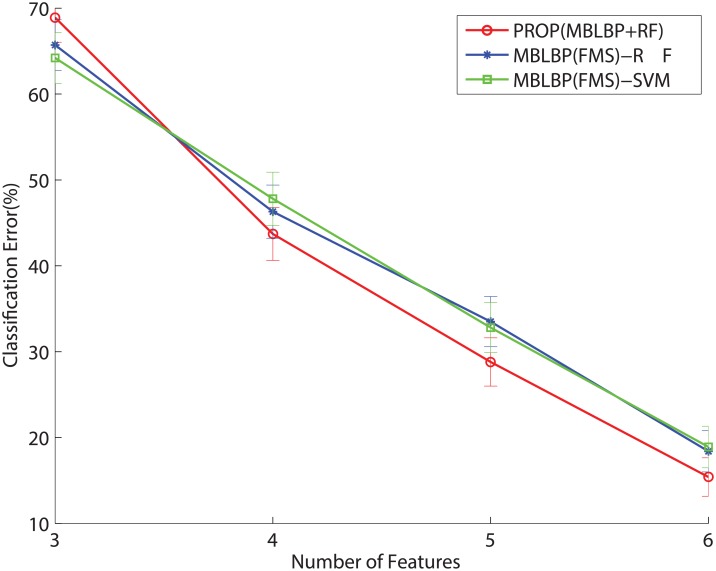
Performance changes with respect to the number of the MB-LBP feature in 7-pose case, using different features selectors and classifiers.

We evaluate the classification accuracies with various feature selections. The classification performance relies on choices of feature subsets to avoid significant loss. The conventional feature selection usually goes through two independent procedures: a filtering process based on independent criteria of supervised learning and an embedding process to choose the best features subset [50]. In the proposed technique, the two steps are jointly combined with the random forest where each node tries to determines the best subset of the MB-LBP features and associated parameters in Eq (6) during the training. Figs 18 and 19 show the classification error rates with the number of features, determined by the different classifiers and feature selection methods. We observe the performance with respect to the number of the chosen features in the 3-pose and the 7-pose cases. The original number of the features is 6 since *k* denotes the *x* − *y* coordinate in an image. We leave *k* out of the feature selection as the MB-LBP is a local feature, so the number of the feature varies from 6 to 3. In Fig 18 that “PROP” denotes the use of the proposed technique while restricting the maximal number of the features. “MBLBP(FMS)+RF” and “MBLBP(FMS)+SVM” denote the compared algorithms, using the independent procedures to choose the features. We apply Fisher-Markov Selector (FMS) with a linear polynomial order [51] as an explicit feature selector to the MB-LBP feature, followed by the random forest and SVM. It is observed in Fig 18, the “PROP” shows only the slight improvements over the two other algorithms. However, when the number of the class increase to 7 in Fig 19, the differences are more visible. That is because the proposed technique performs the joint optimization during the feature selection. The FMS is a generic feature selector, but it works well when the number of the features is much greater than the number of the classes [51].

#### 4.2.4 Performance analysis in inter-data base experiments

In this subsection, we show the results of inter-data base experiments. The parameters in the random forest are trained with the MultiPIE data, and then the model is tested with different data bases such as AFLW, AFW, 300W, and Pointing04 [42–45]. As 300W and AFW have smaller facial samples, we merge the same number of samples from the two data-bases into one named “AFW&300W” in the evaluation.

**Fig 20:**
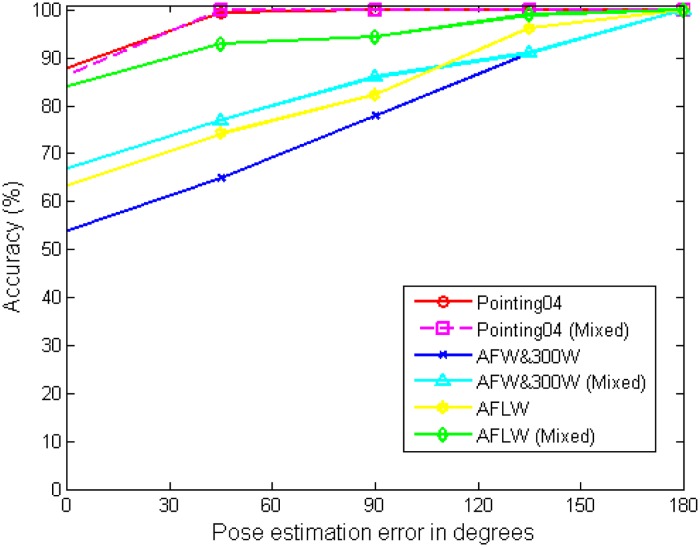
Cumulative head pose estimation error (%) of test images with respect to a degree in inter-db experiments.

Fig 20 shows the cumulative head pose estimation error (%) distributions using the wild data bases, denoted by “Pointing04”, “AFW&300W”, and “AFLW”. The proposed technique provides fairly good performance when using in-the wild data bases such as “AFW&300W” and “AFLW” but also provides comparable results with the intra-database experiments in “Pointing04.” Pointing04 data base is acquired in laboratory condition as in MultiPIE. Thus, the performance is similar to one another. In Fig 20, “Pointing04 (Mixed)”, “AFW&300W (Mixed)”, and “AFLW (Mixed)” show the results when the training samples are evenly chosen from MultiPIE data base and the wild data bases and the testing samples are chosen solely from the corresponding wild data bases. As shown, the performance increases significantly, especially in “AFW&300W” and “AFLW”. Tables 3 and 4 shows the classification errors (CE), the mean absolute errors (ME) of the degrees, and the standard deviation (STD) of the compared algorithms in inter-bases experiments and in mixed inter-bases experiments. According to the results, the proposed technique achieves the best performance among the compared algorithms. The random forest is used in the proposed technique, Kim *et al*. [22], and Drouard *et al*. [24] while the other three techniques [13, 19, 21] use the support vector machine. It is observed that the techniques using the random forest provides much better performance in the inter-data base cases.

**Table 3:** The classification errors (CE)%, the mean absolute errors (MAE) in degree of the proposed technique, and the standard deviation (STD) of the degrees in inter-bases experiments.

Database	Pointing04			AFLW			AFW&300W		
Method	CE	MAE	STD	CE	MAE	STD	CE	MAE	STD
Ma [21]	17.1	10.4	13.6	58.9	76.5	58.2	70.3	79.2	58.5
Kim [22]	15.2	7.1	8.3	40.1	37.2	27.1	50.7	53.9	48.2
Drouard [24]	14.6	6.5	5.8	41.5	38.4	24.0	47.2	52.8	31.1
Yang [19]	21.6	16.9	12.7	56.3	70.5	63.8	68.3	75	63.1
Saeed [13]	18.7	13.0	15.9	18.7	13.0	17.4	60.8	71.6	50.5
Proposed(OL)	13.5	6.1	8.2	36.6	34.8	27.5	46.4	48.6	32.9

**Table 4:** The classification errors (CE)%, the mean absolute errors (MAE) in degree of the proposed technique, and the standard deviation (STD) of the degrees in mixed inter-bases experiments.

Database	Pointing04			AFLW			AFW&300W		
Method	CE	MAE	STD	CE	MAE	STD	CE	MAE	STD
Ma [21]	15.7	7.3	12.4	27.3	36.0	25.6	44.6	52.3	32.8
Kim [22]	13.6	5.8	8.2	19.5	8.9	13.7	36.1	38.0	24.8
Drouard [24]	13.2	5.3	7.4	18.6	8.3	9.6	38.2	40.3	29.5
Yang [19]	16.2	8.6	10.3	29.6	37.4	26.6	51.9	53.1	38.7
Saeed [13]	17.3	10.8	8.6	28.5	36.9	29.5	42.8	50.6	36.7
Proposed(OL)	11.2	2.8	4.7	16.0	6.1	7.8	33.0	35.6	28.6

## 5 Conclusion

We proposed an efficient head pose estimation technique using random forest and texture analysis including gaussian pyramid, multi-scaled block LBP features. In the proposed technique a randomized tree with the feature parameters was trained to yield the improved accurate estimation performance. The features were used at each node for maximizing an information gain, and as a result, the distribution of a particular class of samples was compact in a leaf node. An efficient split function was also developed for each sample to efficiently traverse the tree. When making a decision, we use a Maximum-A-Posteriori criterion for determining the classes of the poses. In the experimental results, the proposed technique showed significantly improved classification performance in the head pose estimation in the various conditions of illumination and occlusions. In the future work, we plan to extend the key idea of the proposed technique to the deep learning framework.
